# Prenatal Stress due to a Natural Disaster Predicts Adiposity in Childhood: The Iowa Flood Study

**DOI:** 10.1155/2015/570541

**Published:** 2015-03-19

**Authors:** Kelsey N. Dancause, David P. Laplante, Kimberly J. Hart, Michael W. O'Hara, Guillaume Elgbeili, Alain Brunet, Suzanne King

**Affiliations:** ^1^Université du Québec à Montréal (UQAM), Montreal, QC, Canada H2X 1Y4; ^2^Douglas Hospital Research Centre, Montreal, QC, Canada H4H 1R3; ^3^University of Illinois at Chicago, Chicago, IL 60607, USA; ^4^University of Iowa, Iowa City, IA 52242, USA; ^5^McGill University, Montreal, QC, Canada H3A 0G4

## Abstract

Prenatal stress can affect lifelong physical growth, including increased obesity risk. However, human studies remain limited. Natural disasters provide models of independent stressors unrelated to confounding maternal characteristics. We assessed degree of objective hardship and subjective distress in women pregnant during severe flooding. At ages 2.5 and 4 years we assessed body mass index (BMI), subscapular plus triceps skinfolds (SS + TR, an index of total adiposity), and SS : TR ratio (an index of central adiposity) in their children (*n* = 106). Hierarchical regressions controlled first for several potential confounds. Controlling for these, flood exposure during early gestation predicted greater BMI increase from age 2.5 to 4, as well as total adiposity at 2.5. Greater maternal hardship and distress due to the floods, as well as other nonflood life events during pregnancy, independently predicted greater increase in total adiposity between 2.5 and 4 years. These results support the hypothesis that prenatal stress increases adiposity beginning in childhood and suggest that early gestation is a sensitive period. Results further highlight the additive effects of maternal objective and subjective stress, life events, and depression, emphasizing the importance of continued studies on multiple, detailed measures of maternal mental health and experience in pregnancy and child growth.

## 1. Introduction

Researchers and public health officials have long recognized the role of maternal health during pregnancy in shaping the health of the infant. In the last three decades, research in the developmental origins of health and disease has highlighted effects extending well beyond infancy [1, 2]. Children whose mothers had poor nutrition during pregnancy are more likely to be born small and have greater risk for obesity and diabetes, particularly if they have rapid growth in the first weeks [3] or months [4, 5] of life. In addition to the prenatal nutritional environment, prenatal stress is increasingly recognized to contribute to cardiometabolic disease risk [6], including later obesity [7] and features of diabetes [8, 9]. This likely reflects effects of maternal stress hormones which, at high levels, can cross the placental barrier and affect fetal development [10], as well as epigenetic changes in the placenta and fetus [11–13]. In addition to adverse effects on fetal growth, which is an independent risk factor for obesity [14], maternal stress might influence long-term metabolic outcomes through effects on the developing hypothalamic pituitary adrenal axis [12, 15] or on metabolism at the cellular level [16] and thereby increase obesity risk independent of effects on birth weight [17].

Despite this growing body of evidence, studies of the effects of prenatal stress on physical growth in humans remain limited. Danish National Register studies indicated that bereavement due to death of a close relative during or shortly before pregnancy was associated with increased risk of overweight among the women's children from ages 10 to 13 years [18] and in early adulthood [19]. Similarly, results from Project Viva, a prospective cohort study of pregnant women and their children, indicated smaller body size but greater central adiposity at age 3 years in association with antenatal depression [20] and with 2nd trimester maternal corticotropin-releasing hormone (CRH), which provides a marker of fetal glucocorticoid exposure [21]. However, a Danish National Birth Cohort study examining a combined measure of maternal distress in pregnancy, reflecting self-reported anxious, depressive, or stress symptoms, showed no associations with offspring overweight at age 7 [22]. These results highlight some of the difficulties of designing human studies of prenatal stress: effects might differ for stress, anxiety, depression, or hormonal markers of stress. Furthermore, anxiety, depression, and bereavement might be associated with one another and with other maternal characteristics that can influence child development. Finally, the effects of prenatal stress on later growth and development depend on the timing of exposure during gestation [23, 24], but many human studies are not able to evaluate the timing of the stressor with accuracy. Thus, we need more studies examining the effects of independent stressors during pregnancy on body composition in children.

Natural disasters provide excellent opportunities to examine the effects of prenatal stress on childhood outcomes because the stressors are independent of potentially confounding genetic and medical risk factors and are relatively randomly distributed with regard to household and maternal characteristics. Furthermore, because the dates of the events are clearly known, we can identify the timing of stress exposure during pregnancy [25]. Our first prospective longitudinal study of prenatal stress due to a natural disaster, Project Ice Storm, has followed the development of children whose mothers were pregnant during a severe ice storm in 1998. Exposure to the ice storm was associated with shorter length at birth [26] and with measures of physical growth later in childhood. Greater objective hardship due to the storm predicted greater body mass index (BMI) and increased risk of obesity at age 5.5 [27], as well as insulin secretion and BMI in adolescence [28]. However, this study left unanswered questions about the effects of prenatal stress on physical growth in early childhood.

In June 2008, an opportunity to replicate Project Ice Storm arose when the U.S. Midwest experienced its worst flooding in more than 50 years. We recruited women exposed to the floods during pregnancy, assessed their stress levels soon after the floods, and collected anthropometric measurements among their children at ages 2.5 and 4 years. We examined relationships between timing and severity of flood exposure and these body composition measurements.

## 2. Materials and Methods

All phases of this study were approved by the University of Iowa Institutional Review Board.

### 2.1. Participants

Immediately following the start of the flooding, we recontacted women enrolled in an existing study of maternal characteristics and pregnancy outcomes at the University of Iowa [29], who had initially been recruited at <20 weeks of gestation from the University of Iowa Hospitals and Clinics. We recruited additional women from three severely flood-affected counties (Linn, Johnson, and Blackhawk). All women were of age 18 or older and English speaking. Of 323 women approached, 268 provided information concerning the flood; 217 were pregnant at the time of the floods.

Families were invited to participate in assessments of children's behavioral, cognitive, and physical outcomes when children were 2.5 and 4 years of age. At age 2.5, 131 families participated in assessments: 27 completed only postal questionnaires and 104 completed face-to-face assessments, when anthropometric measurements were collected. At age 4, 105 families participated in assessments: 24 completed only postal questionnaires and 81 completed face-to-face assessments.

Anthropometric data were missing for some participants, leaving a final sample of 106 women who were exposed to the floods in the 3rd (*n* = 34), 2nd (*n* = 41), or 1st (*n* = 31) trimester and their children (58 boys, 48 girls) who participated in the assessments at age 2.5 only (*n* = 29), age 4 only (*n* = 7), or both ages (*n* = 70).

### 2.2. Assessments

Anthropometric measurements were collected following standard guidelines [30]. Standing height was measured without shoes to the nearest 0.1 cm and weight to the nearest 0.1 kg for mothers and children. Children's triceps and subscapular skinfolds were measured three times each on the right side of the body using Lange calipers, and the mean of the three measurements was used for analyses.

### 2.3. Control Variables

At recruitment in 2008, we collected demographic information; maternal medical and obstetric history; and information on smoking (number of cigarettes/day) and alcohol consumption (number of drinks/week) during pregnancy, using telephone interviews and mail questionnaires. Socioeconomic status (SES) was determined based on parental education and occupation status using the Hollingshead Social Position Criteria [31]. Medical and obstetric history variables relevant to the tested outcomes were combined into an obstetric/fetal risk factor variable, which included history of kidney disease; hypertension; anemia; heart disease; seizures; diabetes; HIV; Rh negative status; asthma; sexually transmitted infections; abnormal blood clotting; thyroid disorders; vaginal, cervical, or urinary tract infections; endocrine disorders; abnormal pregnancy weight gain (<4 kg or >18 kg); preeclampsia; or abnormal bleeding during pregnancy.

Twelve months after the flood, we assessed stressful maternal life events (other than the flood) using the Life Experiences Survey (LES) [32], a self-report measure of life changes, such as death of a spouse or a work promotion. Women were instructed to indicate events occurring from the beginning of their 2008 pregnancy up to the present day. Mothers also completed the Inventory of Depression and Anxiety Symptoms (IDAS) [33], a self-report measure of depression and anxiety symptoms.

At the 2.5-year assessments, we collected data on breastfeeding patterns using semistructured interviews, during which women recalled the age and duration of exclusive, predominant, and mixed breastfeeding, as well as the age of introduction of other foods.

### 2.4. Flood-Related Variables

#### 2.4.1. Objective Hardship

We assessed the severity of flood-related events experienced by participants using a questionnaire that tapped into four categories used in other disaster studies: Threat, Loss, Scope, and Change [34]. Because each natural disaster presents unique experiences, questions must be tailor-made. Our scale included questions specific to the flood, such as days without electricity, damage to the home, and danger due to flood waters. Each dimension was scored on a scale of 0–25 ranging from no exposure to high exposure. A total score (IF100) was calculated by summing the four dimensions using McFarlane's approach [35]. A detailed presentation of the scale is presented elsewhere [36]. In the present sample, scores ranged from 0 to 50 out of a possible 100 points.

#### 2.4.2. Subjective Distress

We assessed women's psychological reaction to the flood using the Impact of Event Scale-Revised (IES-R) [37]. This 22-item scale describes symptoms from 3 categories relevant to posttraumatic stress disorder: intrusions (thoughts and images), hyperarousal, and avoidance. Participants responded on a 5-point Likert scale, from “0-*Not at all*” to “4-*Extremely*,” the extent to which each behavior described how they felt over the preceding 7 days. Items were written to reflect symptoms relative to the flood. The total score was used in analyses. In the present sample, scores ranged from 0 to 60 out of a possible 88 points.

#### 2.4.3. Timing of Exposure

The timing of flood exposure during pregnancy was defined as the number of days between June 15, 2008—the peak of the floods—and the infant's due date. Third trimester exposure corresponds to due dates falling between 0 and 93 days following June 15th; 2nd trimester, 94–186 days; and 1st trimester, 187–279 days.

### 2.5. Outcome Variables

Outcome variables included sex- and age-specific body mass index *Z*-scores based on Center for Disease Control (CDC) child growth standards [38]; subscapular and triceps skinfold sum (SS + TR), an index of total adiposity [39]; and subscapular to triceps skinfold ratio (SS : TR), an index of central adiposity [39].

### 2.6. Statistical Analyses

Objective hardship (IF100) and subjective distress (IES-R) scores were right-skewed and were thus log-transformed for analysis. In addition to child sex, eight covariates expected to be potentially related to child outcomes based on the literature reviews were included in analyses: birth weight (g), obstetric/fetal risk score, maternal BMI (measured during the 2.5-year assessments for analyses of child outcomes at age 2.5 and for the difference between ages 2.5 and 4 and measured during the 4-year assessments for analyses of child outcomes at age 4), smoking during pregnancy (per day), breastfeeding duration (months), SES, general depression, and number of life events.

We tested relationships between predictor and outcome variables using hierarchical linear regression. In a series of individual steps, we first entered child sex and control variables, followed by flood variables: exposure timing, objective hardship, and subjective distress. In a second set of models, for analyses of SS + TR and SS : TR, we included child BMI *Z*-score in the control variables, measured during the 2.5-year assessments for analyses at age 2.5 and for the difference between ages 2.5 and 4 and measured during the 4-year assessments for analyses at age 4. Finally, in a third set of models, we entered interactions after the flood variables, including objective hardship∗sex, subjective distress∗sex, objective hardship∗timing, subject distress∗timing, and objective hardship∗subjective distress. All tests used an* a priori* alpha level of 0.05 (two-sided tests). No measure was taken to correct for multiple testing, as analyses were considered exploratory. Analyses were conducted with SPSS 20.0.

## 3. Results

### 3.1. Sample Characteristics and Correlations

Table 1 presents correlations among all study variables as well as their means and standard deviations. Significant correlations suggested that greater objective hardship (IF100) predicted a greater increase in total adiposity between ages 2.5 and 4. Greater subjective maternal distress (IES-R) predicted greater total adiposity at both 2.5 and 4 years. Timing of the floods earlier in gestation predicted greater BMI at age 4, a greater increase in BMI between the two assessments, and greater total adiposity at ages 2.5 and 4 years. Several control variables were also significantly correlated with outcomes. There were no mean differences in predictor variables (flood variables or covariates) among participants who were measured at only age 2.5, only age 4, or both ages (data not shown).

### 3.2. Multiple Linear Regression Models

Results of regression analyses for each outcome variable are shown in Tables 2–4 and show the progression of variance explained (*R* ^2^) with each step.

### 3.3. Body Mass Index (BMI) *Z*-Scores (Table 2)

#### 3.3.1. Age 2.5

At entry into the model, birth weight (*P* = 0.03) and maternal BMI (*P* = 0.01) predicted child BMI *Z*-scores. In the final model, larger birth weight (*P* = 0.03), fewer fetal risk factors (*P* = 0.01), and larger maternal BMI (*P* = 0.03) predicted greater BMI *Z*-scores. There were no effects of severity of objective hardship or subjective distress due to flood exposure nor of the timing of flood exposure.

#### 3.3.2. Age 4

At entry into the model, fetal risk factors (*P* = 0.04) and maternal BMI (*P* < 0.01) predicted child BMI *Z*-scores. In the final model, fetal risk factors did not retain significance (*P* = 0.56). Larger maternal BMI (*P* < 0.01) predicted greater BMI *Z*-scores. There were no effects of the severity or timing of flood exposure.

#### 3.3.3. Difference between Ages 2.5 and 4

At entry into the model, fetal risk factors (*P* < 0.01) and maternal smoking (*P* = 0.04) predicted difference in BMI *Z*-scores. There were no effects of the severity of hardship or distress due to flood exposure. However, earlier timing of exposure (*P* = 0.04) predicted a greater increase in BMI *Z*-scores from age 2.5 to 4. In the final model, fetal risk factors (*P* = 0.09) and smoking (*P* = 0.08) did not retain significance, but earlier timing of flood exposure (*P* = 0.03) predicted a greater increase in BMI *Z*-scores from age 2.5 to 4.

### 3.4. Total Adiposity (SS + TR) (Table 3)

#### 3.4.1. Age 2.5

There were no effects of covariates or of the severity of objective hardship or subjective distress due to the flood on total adiposity. However, at entry into the model (*P* = 0.04) and in the final model (*P* = 0.03), exposure timing predicted total adiposity at age 2.5: earlier timing of exposure predicted greater adiposity. The effects of exposure timing remained significant in the final model (*P* = 0.03) even when controlling for child BMI *Z*-score at age 2.5 (full results not shown).

#### 3.4.2. Age 4

At entry into the model, maternal BMI (*P* < 0.01) and smoking (*P* < 0.01) predicted total adiposity. In the final model, fewer fetal risk factors (*P* = 0.01), larger maternal BMI (*P* < 0.01), and more smoking during pregnancy (*P* < 0.01) predicted greater adiposity. There were no effects of the severity or timing of flood exposure on total adiposity at age 4.

#### 3.4.3. Difference between Ages 2.5 and 4

At entry into the model, smoking (*P* < 0.01) predicted the difference in total adiposity between ages 2.5 and 4. In addition, the severity of both objective hardship (*P* = 0.02) and subjective distress (*P* = 0.04) due to the floods predicted the difference in adiposity between ages 2.5 and 4. In the final model, more smoking during pregnancy (*P* < 0.01), a greater number of maternal life events (*P* = 0.04), greater objective hardship due to the flood (*P* = 0.03), and greater subjective distress due to the flood (*P* = 0.04) all predicted a greater increase in total adiposity. The effects of objective hardship and subjective distress remained significant (*P* = 0.03 and *P* = 0.04, resp.) even when controlling for child BMI *Z*-score at age 2.5 (full results not shown).

### 3.5. Central Adiposity (SS : TR)

#### 3.5.1. Age 2.5

There were no effects of covariates or of the severity of objective hardship or subjective distress due to the flood on central adiposity at age 2.5 at entry into the model. In the final model, the fetal risk variable was the only predictor of central adiposity: fewer fetal risk factors predicted greater central adiposity (*P* = 0.04). There were no effects of the timing or severity of flood exposure.

#### 3.5.2. Age 4

At entry into the model and in the final model, maternal general depression was the only predictor of central adiposity (at entry, *P* = 0.05; final model, *P* = 0.04). There were no effects of the timing or severity of flood exposure. The effects of maternal depression remained significant in the final model (*P* = 0.05) even when controlling for BMI *Z*-scores at age 4 (full results not shown).

#### 3.5.3. Difference between Ages 2.5 and 4

At entry into the model, birth weight (*P* = 0.05) and maternal general depression (*P* = 0.01) predicted the difference in central adiposity between ages 2.5 and 4. There were no effects of the timing or severity of flood exposure. In the final model, greater maternal depression predicted greater central adiposity (*P* = 0.02); birth weight did not retain significance (*P* = 0.16). The effects of maternal depression remained significant in the final model (*P* = 0.03) even when controlling for BMI *Z*-scores at age 2.5 (full results not shown).

There were no effects of interaction terms in any model (results not shown). In all analyses, variance inflation factors (VIF) were low (less than 2.8) indicating that results were not affected by multicollinearity among variables.

## 4. Discussion

Our results indicate that exposure to a natural disaster during early gestation predicts greater total adiposity at age 2.5 and a greater increase in BMI *Z*-scores from age 2.5 to 4. These results suggest that early pregnancy is a sensitive period for the effects of prenatal stress on childhood growth. Furthermore, prenatal objective hardship and subjective distress exposure significantly and independently predicted a greater increase in total adiposity from age 2.5 to 4 years; a greater number of stressful maternal life events (other than the flood) before and during pregnancy predicted this increase independently of the flood variables. Timing of flood exposure in pregnancy, objective hardship, and subjective distress together increased variance explained by up to 10% over and above that explained by covariates. This supports other studies suggesting that prenatal stress exposure can increase adiposity. Furthermore, our results highlight that effects are evident even in early childhood, which might be a particularly sensitive period for the development of obesity in adulthood [40].

As noted above, the effects of prenatal stress on later growth outcomes might reflect effects on central regulators of metabolism or metabolism at the cellular level, as well as through adverse effects on early growth [17]. The effects of stress exposure in our study persisted even after controlling for birth weight, which supports effects of prenatal stress on central regulators of growth and metabolism rather than through early growth patterns alone.

We observed no effects of flood exposure on central adiposity (SS : TR). However, maternal general depression predicted greater central adiposity at age 4 and a greater increase from age 2.5 to 4. This supports results from Project Viva indicating that antenatal depression predicts greater central adiposity (SS : TR) at age 3 years [20], as well as studies indicating that greater maternal depressive symptoms predict greater risk of overweight in children aged 6–24 months [41]. Maternal depressive symptoms are often associated with adverse maternal health behaviors such as poor diet and exercise patterns, as well as adverse infant and child feeding patterns [41, 42].

Whereas our studies analyze depression at different time points and the mechanisms underlying the effects of prenatal depression are likely to differ from those of postpartum depression, they highlight the importance of maternal depression on adiposity in infancy and the need to distinguish between maternal stress, depression, anxiety, and other measures of maternal mental health in analyses. Differing physiological responses to stress, anxiety, and depression likely result in different mechanistic pathways underlying the effects of each factor on child outcomes [43]; a failure to distinguish between different measures of maternal mental health might obscure effects on child development.

### 4.1. Strengths and Limitations

Our study is limited by the relatively small sample size for some outcomes, which reduces statistical power and limits the analyses we can conduct. Furthermore, parental body size is a major predictor of children's body size. Although we were able to control for maternal BMI, we do not have anthropometric measurements for most of the children's fathers. However, since fathers' BMI is unlikely to be related to the timing or severity of flood exposure, it is unlikely that this introduces systematic bias into our analyses.

The independent nature of the stressor is the major strength of our study. Flood exposure is unlikely to be related to potentially confounding genetic or socioeconomic characteristics that might affect childhood body composition; for example, we found low correlation between objective hardship (IF100) and SES (*r* < 0.20) in the full sample. We were also able, unlike most studies, to tease apart the relative effects of maternal objective hardship and maternal distress to determine their relative effects. The prospective nature of the study is another strength. Our assessments included the measurement of many household and maternal characteristics that might act as confounders. The persistence of the effects of flood exposure, despite the inclusion of these covariates in all analyses, highlights that prenatal stress can independently affect body composition in childhood. Furthermore, these analyses extended results on the effects of maternal general depression on central adiposity, highlighting differences between the effects of maternal stress and maternal depression on childhood body composition and the need for further research.

## 5. Conclusions

Research on the developmental origins of health and disease, originally focused on poor maternal nutrition and later cardiometabolic diseases, now highlights that stress during pregnancy is also important in physical growth patterns and obesity risk [9]. Using the Iowa floods as a stressor, we show that exposure in early pregnancy and both objective and subjective stress are associated with greater adiposity in early childhood and a greater increase with age. With a strong body of the literature now supporting these relationships, we must begin to more precisely differentiate between effects of different aspects of maternal mental health on children's development. This research will complement mechanistic research on epigenetic pathways underlying the effects of maternal stress on children's development [44], with the ultimate goal of improving women's and children's health.

## Figures and Tables

**Table 1:** Correlations among predictor and outcome variables and descriptive statistics.

	1	2	3	4	5	6	7	8	9	10	11	12	Mean	SD	*n*
Predictors															
1 Obj. hardship	1	0.40^**^	0.18	−0.05	−0.15	0.09	−0.01	0.09	−0.06	−0.09	0.18	0.22^*^	1.8	0.8	106
2 Subj. distress	0.40^**^	1	0.00	0.10	−0.02	0.12	0.02	−0.01	−0.26^*^	−0.21^*^	0.30^**^	0.19	1.3	1.1	106
3 Timing	0.18	0.00	1	0.07	−0.02	0.04	−0.08	−0.10	0.10	0.02	−0.17	−0.01	140.5	78.0	106
4 Birth weight	−0.05	0.10	0.07	1	−0.02	0.02	−0.08	−0.05	0.06	0.02	0.09	0.02	3531	469	106
5 Fetal risk	−0.15	−0.02	−0.02	−0.02	1	0.57^**^	0.53^**^	0.06	−0.25^*^	−0.18	−0.11	0.13	0.6	0.9	106
6 Mat. BMI 2.5	0.09	0.12	0.04	0.02	0.57^**^	1	0.92^**^	0.00	−0.40^**^	−0.19	0.06	0.26^**^	26.5	5.8	99
7 Mat. BMI 4	−0.01	0.02	−0.08	−0.08	0.53^**^	0.92^**^	1	0.09	−0.46^**^	−0.20	0.02	0.27^*^	27.3	7.3	75
8 Smoking	0.09	−0.01	−0.10	−0.05	0.06	0.00	0.09	1	0.01	−0.14	0.18	0.25^*^	0.3	1.5	106
9 BF duration	−0.06	−0.26^*^	0.10	0.06	−0.25^*^	−0.40^**^	−0.46^**^	0.01	1	0.26^*^	−0.07	−0.03	8.2	7.0	98
10 SES	−0.09	−0.21^*^	0.02	0.02	−0.18	−0.19	−0.20	−0.14	0.26^*^	1	−0.16	−0.26^**^	53.3	9.8	106
Mat. gen. depr.	0.18	0.30^**^	−0.17	0.09	−0.11	0.06	0.02	0.18	−0.07	−0.16	1	0.42^**^	33.2	8.2	106
Mat. life events	0.22^*^	0.19	−0.01	0.02	0.13	0.26^**^	0.27^*^	0.25^*^	−0.03	−0.26^**^	0.42^**^	1	3.0	2.1	106
Child outcomes															
BMIZ 2.5	0.02	0.17	−0.05	0.24^*^	−0.12	0.14	0.31^*^	0.03	−0.09	−0.09	0.08	−0.01	−0.03	1.00	98
BMIZ 4	−0.15	0.08	−0.24^*^	0.16	0.20	0.45^**^	0.51^**^	−0.03	−0.19	−0.12	0.07	0.14	0.40	1.09	77
BMIZ dif.	0.06	0.01	−0.28^*^	−0.08	0.34^**^	0.29^*^	0.34^**^	0.29^*^	−0.10	−0.05	−0.03	0.16	0.38	0.85	69
SS + TR 2.5	0.09	0.10	−0.25^*^	0.06	−0.12	0.05	0.22	−0.05	−0.10	0.13	0.09	−0.10	13.9	2.7	88
SS + TR 4	0.13	0.26^*^	−0.26^*^	0.09	0.08	0.32^*^	0.42^**^	0.15	−0.24	−0.22	0.04	0.14	17.2	3.7	62
SS + TR dif.	0.38^**^	0.42^**^	−0.18	0.07	0.19	0.28^*^	0.36^*^	0.67^**^	−0.11	−0.16	0.03	0.27	3.3	3.1	52
SS : TR 2.5	0.03	−0.06	0.00	0.00	−0.20	−0.04	−0.01	0.07	0.19	−0.07	−0.07	0.10	0.61	0.17	88
SS : TR 4	−0.11	0.03	−0.13	0.23	−0.08	−0.11	−0.11	0.03	0.15	0.00	0.23	−0.02	0.64	0.22	62
SS : TR dif.	−0.04	0.18	−0.19	0.27	0.02	−0.13	−0.13	0.04	0.11	0.09	0.36^**^	−0.07	0.06	0.26	52

^*^ *P* < 0.05; ^**^ *P* < 0.01. Abbreviations: Obj. hardship: objective hardship; Subj. distr.: subjective distress; Mat.: maternal; BMI: body mass index; BF duration: breastfeeding duration (months); SES: socioeconomic status; gen. depr.: general depression; BMIZ: body mass index *Z*-Score; Dif.: difference between values at age 2.5 and 4 years; SS: subscapular skinfold; TR: triceps skinfold.

**Table 2:** Summary of hierarchical linear regression analyses for body mass index (BMI). Significant effects are indicated in bold.

Predictor variables	Values at entry into the model					Final model		
	*R* ^2^	Δ*R* ^2^	*F*	Δ*F*	Sig. Δ*F*	Unstand. coeff. (*B*)	Stand. coeff. (*β*)	Sig.
Age 2.5								
(Constant)						−2.19		0.10
Sex	0.00	0.00	0.23	0.23	0.63	−0.01	−0.01	0.96
Birth weight	0.05	0.05	2.53	4.81	**0.03**	0.00	0.23	**0.03**
Fetal risk factors	0.07	0.02	2.24	1.63	0.20	−0.39	−0.33	**0.01**
Maternal BMI	0.13	0.06	3.32	6.19	**0.01**	0.05	0.30	**0.03**
Smoking	0.13	0.01	2.76	0.58	0.45	0.06	0.09	0.41
Breastfeeding dur.	0.14	0.00	2.38	0.52	0.47	0.00	−0.01	0.96
Household SES	0.14	0.00	2.08	0.41	0.52	−0.01	−0.08	0.45
Mat. gen. depr.	0.14	0.00	1.80	0.00	0.96	0.00	−0.02	0.86
Mat. life events	0.15	0.00	1.65	0.50	0.48	−0.04	−0.08	0.50
Exposure timing	0.15	0.01	1.53	0.57	0.45	0.00	−0.07	0.49
Obj. hardship	0.15	0.00	1.38	0.03	0.87	−0.08	−0.07	0.56
Subj. distress	0.17	0.01	1.38	1.37	0.25	0.13	0.14	0.25
Age 4								
(Constant)						−3.24		**0.04**
Sex	0.00	0.00	0.06	0.06	0.81	0.01	0.01	0.96
Birth weight	0.03	0.03	0.95	1.84	0.18	0.00	0.16	0.16
Fetal risk factors	0.09	0.06	2.10	4.30	**0.04**	−0.10	−0.07	0.56
Maternal BMI	0.30	0.21	6.76	18.95	**<0.01**	0.11	0.67	**<0.01**
Smoking	0.30	0.00	5.32	0.00	0.99	−0.02	−0.02	0.90
Breastfeeding dur.	0.31	0.01	4.50	0.60	0.44	0.03	0.19	0.14
Household SES	0.31	0.00	3.84	0.19	0.66	0.00	−0.02	0.85
Mat. gen. depr.	0.31	0.00	3.31	0.03	0.86	0.00	0.03	0.83
Mat. life events	0.32	0.01	2.96	0.45	0.50	−0.04	−0.07	0.57
Exposure timing	0.36	0.04	3.17	3.74	0.06	0.00	−0.17	0.13
Obj. hardship	0.39	0.02	3.13	2.15	0.15	−0.29	−0.22	0.07
Subj. distress	0.41	0.03	3.17	2.55	0.12	0.20	0.19	0.12
Difference								
(Constant)						0.13		0.92
Sex	0.00	0.00	0.02	0.02	0.90	0.07	0.04	0.74
Birth weight	0.01	0.01	0.26	0.50	0.48	0.00	0.01	0.93
Fetal risk factors	0.13	0.12	3.18	8.97	**<0.01**	0.26	0.25	0.09
Maternal BMI	0.14	0.01	2.62	0.92	0.34	0.02	0.14	0.37
Smoking	0.20	0.06	3.06	4.28	**0.04**	0.29	0.23	0.08
Breastfeeding dur.	0.20	0.00	2.56	0.25	0.62	0.01	0.10	0.49
Household SES	0.20	0.00	2.16	0.04	0.84	0.00	0.00	0.98
Mat. gen. depr.	0.21	0.01	1.97	0.66	0.42	−0.01	−0.12	0.37
Mat. life events	0.21	0.00	1.73	0.06	0.80	0.03	0.06	0.69
Exposure timing	0.27	0.06	2.09	4.42	**0.04**	0.00	−0.27	**0.03**
Objective hardship	0.28	0.01	1.94	0.58	0.45	0.11	0.11	0.41
Subjective distress	0.28	0.00	1.76	0.17	0.68	−0.05	−0.06	0.68

Abbreviations: BMI: body mass index; Breastfeeding dur.: breastfeeding duration (months); SES: socioeconomic status; Mat.: maternal; Gen. depr.: general depression.

**Table 3:** Summary of hierarchical linear regression analyses for total adiposity (SS + TR). Significant effects are indicated in bold.

Predictor variables	Values at entry into model					Final model		
	*R* ^2^	Δ*R* ^2^	*F*	Δ*F*	Sig. Δ*F*	Unstand. coeff. (*B*)	Stand. coeff. (*β*)	Sig.
Age 2.5								
(Constant)						5.73		0.15
Sex	0.03	0.03	2.51	2.51	0.12	0.98	0.18	0.11
Birth weight	0.04	0.01	1.67	0.83	0.36	0.00	0.14	0.21
Fetal risk factors	0.05	0.01	1.47	1.07	0.30	−0.58	−0.19	0.18
Maternal BMI	0.08	0.03	1.80	2.69	0.10	0.10	0.20	0.17
Smoking	0.08	0.00	1.42	0.02	0.90	−0.03	−0.02	0.87
Breastfeeding dur.	0.09	0.01	1.32	0.80	0.37	−0.02	−0.06	0.63
Household SES	0.12	0.03	1.48	2.34	0.13	0.04	0.15	0.22
Mat. gen. depr.	0.13	0.01	1.41	0.94	0.33	0.04	0.11	0.35
Mat. life events	0.14	0.01	1.36	0.91	0.34	−0.15	−0.12	0.37
Exposure timing	0.18	0.05	1.70	4.24	**0.04**	−0.01	−0.25	**0.03**
Objective hardship	0.20	0.02	1.70	1.60	0.21	0.43	0.14	0.28
Subjective distress	0.20	0.00	1.54	0.06	0.80	0.08	0.03	0.80
Age 4								
(Constant)						9.13		0.10
Sex	0.00	0.00	0.02	0.02	0.90	−0.21	−0.03	0.82
Birth weight	0.00	0.00	0.11	0.21	0.65	0.00	0.00	0.98
Fetal risk factors	0.02	0.02	0.33	0.78	0.38	−2.06	−0.43	**0.01**
Maternal BMI	0.24	0.22	3.78	13.85	**<0.01**	0.31	0.61	**<0.01**
Smoking	0.47	0.23	8.29	20.27	**<0.01**	4.80	0.45	**<0.01**
Breastfeeding dur.	0.47	0.00	6.79	0.09	0.76	0.03	0.06	0.67
Household SES	0.47	0.00	5.70	0.02	0.89	0.00	0.01	0.94
Mat. gen. depr.	0.47	0.00	4.89	0.05	0.82	0.01	0.01	0.94
Mat. life events	0.47	0.00	4.28	0.15	0.70	−0.16	−0.07	0.60
Exposure timing	0.48	0.01	3.92	0.84	0.37	−0.01	−0.11	0.36
Objective hardship	0.49	0.00	3.52	0.22	0.64	0.02	0.00	0.97
Subjective distress	0.52	0.03	3.57	2.63	0.11	0.73	0.21	0.11
Difference								
(Constant)						−1.15		0.77
Sex	0.02	0.02	0.99	0.98	0.33	−0.12	−0.02	0.86
Birth weight	0.02	0.00	0.58	0.18	0.67	0.00	0.07	0.47
Fetal risk factors	0.05	0.03	0.89	1.49	0.23	−0.65	−0.16	0.30
Maternal BMI	0.10	0.05	1.29	2.42	0.13	0.07	0.14	0.33
Smoking	0.54	0.44	10.74	43.84	**<0.01**	5.41	0.60	**<0.01**
Breastfeeding dur.	0.54	0.00	8.78	0.06	0.80	0.05	0.11	0.34
Household SES	0.54	0.00	7.38	0.07	0.79	−0.01	−0.02	0.83
Mat. gen. depr.	0.55	0.01	6.45	0.52	0.47	−0.08	−0.19	0.07
Mat. life events	0.57	0.03	6.28	2.77	0.10	0.44	0.23	**0.04**
Exposure timing	0.58	0.00	5.55	0.15	0.70	0.00	−0.08	0.42
Objective hardship	0.63	0.06	6.20	5.96	**0.02**	0.94	0.23	**0.03**
Subjective distress	0.67	0.04	6.54	4.45	**0.04**	0.71	0.23	**0.04**

Abbreviations: BMI: body mass index; Breastfeeding dur.: breastfeeding duration (months); SES: socioeconomic status; Mat.: maternal; Gen. depr.: general depression.

**Table 4:** Summary of hierarchical linear regression analyses for central adiposity (SS : TR). Significant effects are indicated in bold.

Predictor variables	Values at entry into model					Final model		
	*R* ^2^	Δ*R* ^2^	*F*	Δ*F*	Sig. Δ*F*	Unstand. coeff. (*B*)	Stand. coeff. (*β*)	Sig.
Age 2.5								
(Constant)						0.76		**<0.01**
Sex	0.00	0.00	0.42	0.41	0.52	0.03	0.09	0.42
Birth weight	0.00	0.00	0.21	0.00	0.96	0.00	0.02	0.83
Fetal risk factors	0.04	0.04	1.22	3.24	0.08	−0.06	−0.30	**0.04**
Maternal BMI	0.05	0.01	1.03	0.47	0.50	0.00	0.15	0.34
Smoking	0.06	0.01	1.02	0.98	0.32	0.01	0.12	0.34
Breastfeeding dur.	0.09	0.03	1.28	2.50	0.12	0.00	0.20	0.14
Household SES	0.11	0.02	1.34	1.66	0.20	0.00	−0.17	0.17
Mat. gen. depr.	0.13	0.03	1.50	2.39	0.13	0.00	−0.20	0.10
Mat. life events	0.14	0.01	1.38	0.50	0.48	0.01	0.10	0.46
Exposure timing	0.14	0.00	1.25	0.23	0.63	0.00	−0.05	0.65
Objective hardship	0.14	0.00	1.12	0.00	0.99	0.00	0.00	0.97
Subjective distress	0.14	0.00	1.02	0.01	0.91	0.00	−0.01	0.91
Age 4								
(Constant)						0.00		1.00
Sex	0.01	0.01	0.72	0.72	0.40	0.04	0.08	0.60
Birth weight	0.08	0.07	2.31	3.85	0.06	0.00	0.21	0.17
Fetal risk factors	0.08	0.00	1.52	0.02	0.89	−0.02	−0.08	0.69
Maternal BMI	0.09	0.01	1.21	0.36	0.55	0.00	−0.01	0.96
Smoking	0.11	0.02	1.20	1.14	0.29	0.08	0.11	0.50
Breastfeeding dur.	0.12	0.01	1.08	0.55	0.46	0.00	0.14	0.46
Household SES	0.13	0.00	0.93	0.14	0.71	0.00	0.01	0.95
Mat. gen. depr.	0.20	0.07	1.36	3.93	**0.05**	0.01	0.33	**0.04**
Mat. life events	0.20	0.00	1.20	0.13	0.72	−0.01	−0.06	0.71
Exposure timing	0.23	0.03	1.29	1.85	0.18	0.00	−0.19	0.22
Objective hardship	0.24	0.00	1.17	0.23	0.63	−0.02	−0.06	0.72
Subjective distress	0.24	0.00	1.06	0.13	0.72	−0.01	−0.06	0.72
Difference								
(Constant)						−0.60		0.19
Sex	0.00	0.00	0.08	0.08	0.78	0.02	0.03	0.81
Birth weight	0.07	0.07	1.98	3.89	**0.05**	0.00	0.20	0.16
Fetal risk factors	0.08	0.00	1.36	0.18	0.67	0.10	0.29	0.19
Maternal BMI	0.11	0.03	1.48	1.79	0.19	−0.01	−0.24	0.26
Smoking	0.11	0.00	1.16	0.00	1.00	−0.12	−0.16	0.35
Breastfeeding dur.	0.12	0.00	0.98	0.15	0.70	0.01	0.15	0.36
Household SES	0.12	0.00	0.82	0.01	0.94	0.00	0.05	0.73
Mat. gen. depr.	0.24	0.12	1.68	6.94	**0.01**	0.01	0.37	**0.02**
Mat. life events	0.25	0.01	1.54	0.53	0.47	−0.02	−0.12	0.45
Exposure timing	0.30	0.06	1.80	3.36	0.07	0.00	−0.27	0.07
Objective hardship	0.31	0.00	1.61	0.16	0.69	0.01	0.03	0.82
Subjective distress	0.33	0.03	1.64	1.61	0.21	0.05	0.20	0.21

Abbreviations: BMI: body mass index; Breastfeeding dur.: breastfeeding duration (months); SES: socioeconomic status; Mat.: maternal; Gen. depr.: general depression.
